# Nosode Cases

**Published:** 1892-11

**Authors:** J. T. Tapley

**Affiliations:** Student, Marysville, Cal.


### NOSODE CASES.

J. T. TAPLEY, STUDENT, MARYSVILLE, CAL.

CASE I.—Miss N. B., a beautiful girl, aged thirteen, was dangerously ill with diphtheria. The two attending physicians—an allopath and an eclectic—were so sure that she was dying that they did not hesitate to express that opinion to several acquaintances of the writer. The priest had administered the last rites of the Church. The grief-stricken parents, who had only the day before buried a beloved son, a valued young man, aged twenty-one years, he having died of the same terrible disease, had given up all hope. At midnight when the feet and limbs and ears were cold and clammy, when it seemed to those who were watching at the bedside that life was fast ebbing away, the father placed in the mouth a powder of Diphtherinum$^{cm}$. In less than ten minutes the patient was sleeping. She slept for four hours and when she awakened she called for food. In about ten days she was about the house. There remained an affection of the eyes and a paralytic condition of the vocal chords and of the lower extremities. Bell.$^{3x}$ given by the mother cured her throat and eye symptoms in forty-eight hours, notwithstanding one of the wise doctors said that she would become idiotic and would never be able to talk. Secale-cor.$^{cc}$, a few doses, were given for the "sensation as if she had no feet." After the remedy she could walk without support but quite frequently the ankles would turn under her and she would fall to the floor. Another picture of the case seemed to point to Gels., which was given, a single dose of the CM, dry on the tongue. Improvement set in and continued. To-day, fifteen months later, she is a blooming young lady without a symptom.

CASE II.—Lizzie B., a sister, aged eight years. Mother said she had been getting sick for two or three weeks. She was pale, languid, no appetite. Did not sleep well and had to be taken from school. There was a defective sewer in front of the house. She was given a single dose of Pyrogen$^{cmm}$ (Swan), dry, on the tongue, and the way she picked up was astonishing. She needed no more medicine.

CASE III.—Mr. Lumbard. Had an eruption on the right leg. He barked his shin two years ago and the wound refused to heal. In one year's time it had spread from the knee to his instep and was just such a case as was reported by Dr. Ruffe on page 299, of the April number of the *Advance.* One day the writer was asked by the patient, who was a personal friend, if Homœopathy could do anything for him. He was told that if he used local applications and suppressed the eruption, it would probably kill him sooner or later. I then noted down all the symptoms I could get, and gave him a dose of Sul.$^{50m}$ (Fincke). As he could see no change after three weeks, he got Psorin.$^{500}$ (B. & T.) one dose, and as that seemed not to affect the eruption one way or the other after prudent waiting, I took another photo and gave Graph.$^{50m}$ (Fincke) in divided dose, and it was like so much water on a duck's back. Now it is quite possible that one of these remedies might have cured this case if it had been properly handled and the right remedy given in the right potency. I will say in this connection that the remedies given were genuine potencies. Of that there is no possible doubt. Three weeks after the last dose and two months after the first dose of Sul., I obtained some of the scab and a few drops of the blood from the worst part of the sore, and sent it in alcohol to Dr. Swan to be potentized. When the remedy was returned the patient got a divided dose of the DMM. As no change was admitted after six days, a dose of the CMM was given; one dose in six spoonfuls of water, one every two hours. Improvement was then marked, but it seemed to last only about ten days. I waited a week, thinking it would again set in, but as it did not, I gave a dose of Pyrogen$^{cmm}$ (Swan), dry, on the tongue. Again improvement set in, and continued for six weeks. Then another dose of the CMM of the morbose product was given in water, and that is all the medicine he got.

The improvement continued with occasional "let ups" until no sign of the eruption remained, two months after the last dose. A brother of this patient has had a similar eruption on his leg for many years.

CASE IV.—Dr. R———, our homœopathic physician, had been treating a young lady suffering with diphtheria in a neighboring town. He told the writer that it was the worst case he had ever seen, and that he believed she would die. He had been giving Phyto, Merc., Peroxide of Hydrogen, Carb-v., etc.

He was given some Diphtherinum$^{cm}$, which he gave to the patient, and she got well right away. The regular (?) who was also looking after the case wanted some of the remedy to have it analyzed, and he is now reading up Homœopathy. This patient died six weeks later of remittent fever.

Two doses of Medorrh$^{50m}$ brought back a profuse, yellow, stinking discharge on a young man who had his "clap" suppressed, seven years after. If necessary, he will take a dose prepared from the same discharge and results will be reported.

